# Socioeconomic position and self-rated health among female and male adolescents: The role of familial determinants in explaining health inequalities. Results of the German KiGGS study

**DOI:** 10.1371/journal.pone.0266463

**Published:** 2022-04-07

**Authors:** Petra Rattay, Miriam Blume, Benjamin Wachtler, Lina Wollgast, Jacob Spallek, Stephanie Hoffmann, Lydia Sander, Raphael Herr, Max Herke, Marvin Reuter, Anna Novelli, Claudia Hövener

**Affiliations:** 1 Department of Epidemiology and Health Monitoring, Robert Koch-Institute, Berlin, Germany; 2 Department of Public Health, Brandenburg University of Technology Cottbus-Senftenberg, Senftenberg, Germany; 3 Medical Faculty Mannheim, Mannheim Institute of Public Health, Social and Preventive Medicine, Heidelberg University, Mannheim, Germany; 4 Medical Faculty, Institute of Medical Sociology, Martin-Luther-University Halle-Wittenberg, Halle (Saale), Germany; 5 Medical Faculty, Institute of Medical Sociology, Centre for Health and Society, Heinrich Heine University Düsseldorf, Düsseldorf, Germany; 6 Chair of Health Economics, Technical University of Munich, Munich, Germany; University of Jyvaskyla, FINLAND

## Abstract

**Objective:**

Although health inequalities in adolescence are well documented, the underlying mechanisms remain unclear. Few studies have examined the role of the family in explaining the association between the family’s socioeconomic position and adolescents’ self-rated health. The current study aimed to explore whether the association between socioeconomic position and self-rated health was mediated by familial determinants.

**Methods:**

Using data from wave 2 of the”German Health Interview and Examination Survey for Children and Adolescents” (KiGGS) (1,838 female and 1,718 male 11- to 17-year-olds), linear regression analyses were conducted to decompose the total effects of income, education, occupational status, socioeconomic position index and adolescents’ subjective social status on self-rated health into direct effects and indirect effects through familial determinants (family cohesion, parental well-being, parental stress, parenting styles, parental obesity, smoking and sporting activity).

**Results:**

A significant total effect of all socioeconomic position indicators on self-rated health was found, except for income in male adolescents. In female adolescents, more than 70% of the total effects of each socioeconomic position indicator were explained by familial mediators, whereas no significant direct effects remained. The most important mediator was parental well-being, followed by family cohesion, parental smoking and sporting activity. In male adolescents, the associations between income, parental education, the socioeconomic position index and subjective social status were also mediated by familial determinants (family cohesion, parental smoking, obesity and living in a single-mother family). However, a significant direct effect of subjective social status remained.

**Conclusion:**

The analysis revealed how a family’s position of socioeconomic disadvantage can lead to poorer health in adolescents through different family practices. The family appears to play an important role in explaining health inequalities, particularly in female adolescents. Reducing health inequalities in adolescence requires policy interventions (macro-level), community-based strategies (meso-level) and programs to improve parenting and family functioning (micro-level).

## Introduction

Health inequality in adolescence is defined as the association between the socioeconomic position (SEP) of the family and adolescents’ health, and has been documented for many years [1–4]. However, the mechanisms underlying this effect remain unclear. The family, as the primary socialization agent, plays a central role in health development in childhood and throughout adolescence [5]. Health-related socialization occurs through everyday family practices and family interactions, through which children and adolescents acquire fundamental attitudes, skills and knowledge that contribute significantly to their mental, physical, social and cognitive development, and thus promotes or hinders healthy development [6]. By “doing family” [7], young people learn from early childhood to adolescence how to deal with their bodies, health and illness, and how to develop coping strategies for everyday life and stress [5]. Moreover, young people are located in a hierarchically structured society through the family, which is accompanied by unequal life chances and opportunities to experience a life involving satisfactory well-being and good health. However, in adolescence, achieving emotional independence and autonomy from parents and the family of origin is a central developmental task [8]. Even in this stage of life, the family has an important mediating function between the granting of security and reliability on the one hand and the granting of autonomous development on the other hand [9,10]. In addition, adolescents’ standing in the social hierarchy continues to be largely determined by the SEP of their parents [9].

Considering this background, the current study aimed to analyze the role of familial determinants through which a family’s position of socioeconomic disadvantage leads to poor general health in adolescents. Regarding the general health of adolescents, self-rated health (SRH) is an important global and valid indicator [11]. SRH is the subjective perception of general health status, and covers the physical, mental and social dimensions of health and well-being [11–13].

Associations between the SEP of the family (determined by income, education and occupational status of the parents) and the health of adolescents have been reported for numerous health outcomes [1,14–17]. Regarding SRH in adolescence, several previous studies have found associations with different indicators of the SEP of the family [14,16,18–23]. However, focusing on health inequalities throughout the life course, the hypothesis regarding health equalization in youth proposed by West [24,25] has been influential in health science. According to this hypothesis, in adolescence—in contrast to childhood and later phases of life—the SEP of the family becomes less important to health because of detachment from parents, while school, peers and the adolescents’ own perception of their social position have an increasingly strong influence on health. The evidence for this theory is mixed [17,24–27], and clear conclusions have not been drawn regarding SRH [17,23,28,29]. Against this background, the analysis of associations between adolescents’ own social status and health has gained importance in recent years, on the basis of the assumption that family SEP determined by income, parental education level and parental occupational status has a decreasing influence as adolescents begin to develop their own status positions. Quon and McGrath [30] reported a meta-analysis indicating a positive association between higher subjective social status (SSS) and better health outcomes. This association was strongest for mental health outcomes, followed by SRH [30]. Some previous studies reported that SSS had even stronger effects on health than the family’s SEP [18,30]. SEP and SSS have also been found to be independently associated with general health [18,21,31].

Besides SEP, the health of adolescents is also influenced by a range of other familial determinants. Family structure is a major family determinant of adolescents’ health, and is strongly linked to the financial position of the family household [32]. On the one hand, economic hardship can cause parental stress and conflict, which can result in the separation of the parents and poor adolescents’ health [33]. On the other hand, single parents are exposed to a very high risk of poverty, particularly in Germany [32], because they typically have only one income at their disposal and often work part-time to care for their children. Some previous studies have confirmed that the association between family structure and health is at least partially mediated by the family’s SEP [34,35]. Furthermore, several previous studies have examined the associations between the psychosocial characteristics of the family and SRH, such as family cohesion, parenting behavior, parental support, parent-adolescent relationships and communication [19,36–39]. Moreover, Breidablik et al. [38] reported that low general well-being and low life satisfaction of the mother or father were important parental factors associated with lower SRH in adolescents. In addition, some studies have analyzed the associations between health behavior-related determinants of family and SRH. Parental smoking, smoking in the household and parental obesity were found to be important parental predictors for poor SRH in adolescents [38,40]. Moreover, SEP is linked to several familial determinants [33,41–45], including family structure [39,46], parenting behavior [43,44] and parental stress [45].

To date, few studies have investigated whether health inequalities in adolescents are mediated by familial determinants [47]. A scoping review by Blume et al. [47] revealed that parenting practices, parental mental health, as well as family conflict and distress, were relevant mediators for health inequalities in childhood and adolescence. Regarding mediation by familial determinants, a relatively large number of studies have examined mental health of young people. In contrast, for other health outcomes (e.g., SRH), this issue has received relatively little investigation. Blume et al.’s review [47] identified only three studies that explored whether the association between SEP and young people’s SRH was mediated by familial determinants [22,48,49]. Of the two studies that analyzed mediation through the parent-child relationship, one revealed a significant result [49] whereas the other found no mediating effect [48]. Moor et al. [49] reported that the relationship with the father was an important mediator explaining the association between the family affluence scale and SRH. Salonna et al. explored whether social inequalities in adolescents’ SRH were explained by parental social support [22]. They observed that social support by the father was a particularly strong mediator of the association between family affluence and SRH among both females and males, as well as the association between financial strain and SRH among males [22].

Because research regarding the explanation of health inequalities in adolescents through familial determinants—particularly studies focusing on SRH—is scarce, the aim of the present study was to explore the extent to which health inequalities among 11- to 17-year-olds can be explained by different familial determinants. We explored different indicators of familial SEP: equivalized household income, parental education level and parental occupational status, an index for the SEP constructed from a sum score of all three indicators, as well as adolescents’ subjective assessment of their social status. Regarding the mediators, different determinants of family life were considered, including psychosocial determinants (family cohesion, parental well-being, number of everyday stressors, and parenting style of the mother and father) and health behavior-related determinants (parental smoking, parental sporting activity and obesity of at least one parent). Finally, the strongest predictors of good health in adolescence were determined by simultaneously considering all familial determinants.

The following research questions were examined:

1. Are there associations between family SEP (household income, parental education level, parental occupational status, SEP index and adolescents’ SSS) and SRH among female and male adolescents aged 11–17 years (total effects)?
2. Are the associations between SEP indicators and female and male adolescents’ SRH mediated by familial determinants (psychosocial and health-behavioral)? How strong are the direct effects and the indirect effects regarding the different SEP indicators?
3. Which familial determinants (psychosocial and health-behavioral) explain the associations of the SEP index and the SSS with adolescents’ SRH?
4. Which socioeconomic, psychosocial and health-behavioral determinants show the strongest associations with SRH when considering all determinants at once?

## Material and methods

### Data

The analysis was conducted using data from wave 2 of the cohort study of the “German Health Interview and Examination Survey for Children and Adolescents” (KiGGS). The KiGGS cohort study was carried out by the Robert Koch Institute as part of the health monitoring commissioned by the German Federal Ministry of Health [50].

A total of 17,640 children and adolescents between the ages of 0 and 17 years and their parents participated in the KiGGS baseline study (2003–2006). The baseline survey was carried out as a combined interview and examination survey. The first follow-up (KiGGS Wave 1) took place in 2009–2012 as a telephone survey. The second follow-up (KiGGS Wave 2) was carried out in 2014–2017 as a combined interview and examination survey. All participants of the KiGGS baseline study who agreed to be contacted again were invited to participate in the KiGGS Wave 2 survey, regardless of their participation in KiGGS Wave 1. At the time of KiGGS Wave 2, the age range of the cohort participants was 10–31 years. A total of 10,853 people participated in the survey (61.5% of the initial sample) [50,51].

In KiGGS Wave 2, some questions regarding the family situation were explicitly included for the first time, such as questions about the parenting styles of the mother and father as well as parental well-being. Because the present analysis focused on the mediating effects of these familial determinants, data from KiGGS Wave 2 were used. Our analysis included only participants from KiGGS Wave 2 who were younger than 18 years of age, because the parents reported their own current socio-economic position just for this age group. For the < 18 years age group, KiGGS Wave 2 comprised data from a total of 2,258 female and 2,340 male participants. The analysis was based on data from 1,838 female and 1,718 male adolescents aged 11–17 years for whom complete information on all variables was available (listwise deletion). The sample characteristics are shown in Table 1.

**Table 1:** Description of the sample.

	Female adolescents (n = 1,838)		Male adolescents (n = 1,718)	
Indicator (range)	Mean (weighted)	SD (weighted)	Mean (weighted)	SD (weighted)
**Self-rated health** (0–4)	3.19	0.64	3.24	0.65
**Household income** (1–7)	4.39	1.75	4.31	1.75
**Education level** (1–7)	4.82	1.52	4.74	1.55
**Occupational status** (1–7)	4.49	1.40	4.50	1.37
**SEP index** (3–21)	13.71	3.84	13.55	3.84
**Subjective social status** (1–10)	6.44	1.26	6.49	1.26
**Family cohesion** (1–100)	61.04	16.68	62.29	15.15
**Parental well-being** (0–100)	78.87	13.77	78.70	14.10
**Number of stressors** (0–13)	1.21	1.78	1.26	1.86
	**n (unweighted)**	**% (weighted)**	**n (unweighted)**	**% (weighted)**
**Parenting style (mother)**				
Authoritative	767	40.6	657	38.8
Emotional distancing	278	15.4	312	17.5
Demanding controlling	268	15.5	294	18.7
Permissive	507	27.7	441	24.5
No mother in household	18	0.9	14	0.5
**Parenting style (father)**				
Authoritative	598	31.6	539	31.5
Emotional distancing	211	11.7	177	10.5
Demanding controlling	348	19.0	379	23.2
Permissive	500	26.9	439	23.5
No father in household	181	10.9	184	11.3
**Parental smoking**				
Yes	663	38.9	626	41.5
No	1,175	61.1	1,092	58.5
**Parental obesity**				
Yes	478	28.1	423	27.0
No	1,360	71.9	1,295	73.0
**Parental sporting activity**				
Yes	1,541	81.8	1,441	80.1
No	297	18.2	277	19.9
**Age**				
10–13	809	43.0	735	44.0
14–17	1,029	57.0	983	56.0
**Migration background**				
None	1,554	78.2	1,445	73.4
One-sided	141	9.4	126	10.5
Two-sided	143	12.4	147	16.2

The KiGGS cohort study is subject to strict compliance with the data protection regulations of Germany’s Federal Data Protection Act. The Hannover Medical School ethics committee considered and approved the survey under ethical guidelines (No. 2275–2014). The Federal Commissioner for Data Protection and Freedom of Information in Germany had no objections to the study. Participation in the study was voluntary. Participants and their parents and/or legal guardians were informed about the objectives and content of the study and data protection, and provided written informed consent.

### Variables

#### Outcome variable

Adolescents’ **self-rated health (SRH)** was operationalized using the first Minimum European Health Module (MEHM1) question [12]. The formulation “In general, what would you say your health status is like?” was based on the recommendations of the World Health Organization (WHO) with a five-step answering scale (answer categories: very good, good, fair, bad and very bad). In our statistical analyses, we used the variable as a metric variable, where a value of 0 indicated very bad health and 4 indicated very good health.

#### Predictor variables

We used the following predictor variables: (a) family household income, (b) parental education level, (c) parental occupational status, (d) the SEP index as the sum score of the three single SEP indicators, as well as (e) the adolescents’ SSS. The operationalization of the single SEP indicators as well as the SES index is consistently used in all surveys conducted at the Robert Koch Institute. The operationalized variables were provided for all users in the data sets, which makes it possible to compare results of the surveys [21]. A detailed description of the operationalization of all SEP indicators in the KiGGS study can be found in Lampert et al. [21].

Family **income** was measured by needs-adjusted net household income (equivalized disposable income) [52,53]. Missing values for net household income were imputed through regression imputation using data on parents’ age, level of education and occupational status, as well as regional information of the German Federal Statistical Office regarding mean net household income in the participants’ residential area [54]. This was followed by a distribution-based classification of income groups [21].

Levels of **education** were assessed using the international Comparative Analysis of Social Mobility in Industrial Nations (CASMIN) classification [21,55], ranging from “no degree” (i.e., inadequately completed general education) to “higher tertiary education” [21].

To operationalize the **occupational status** of the parents [21] we used Ganzeboom and Treimann’s International Socio-Economic-Index of Occupational Status (ISEI) [56]. ISEI is derived from the 2008 International Standard Classification of Occupations (ISCO-08) [57].

Each of the three single SEP indicators was transformed to a range of values from 1 (low) to 7 points (high); a detailed tabular representation of the assignment can be found in Lampert et al. [21]. Regarding education level and occupational status, children and adolescents were assigned the maximum point score their parents provided. For cases in which the child lived exclusively with only one parent, the score of that single parent was assigned directly [21]. Lampert et al. [21] proposed a metric scale for the individual SEP dimensions because the intervals between the point scores for all three indicators reflect equidistant intervals regarding external criteria.

The **SEP index** was calculated by the sum of the point scores of all three equally-weighted subscales of household income, education level, and occupational status. SEP index values ranged between 3 and 21, and were used as a household index for all family members [21]. The SEP index can better depict the additive effect of the three single SEP indicators than their simultaneous inclusion in one model. The use of a composite multidimensional SEP index can help to avoid problems caused by the correlated nature of single SEP indicators or their interactive effects [58]. The calculation of a variance inflation factor revealed moderate multicollinearity of the three individual SEP indicators.

Adolescents’ **subjective social status** (SSS) was included to capture the perception of the social situation of the family by the adolescents themselves. The SSS was recorded in KiGGS Wave 2 using a German version of the MacArthur Scale for adolescence developed by Goodman et al. [31,59] and adapted for Germany by Lampert et al. [21]. The scale operates using an image of a 10-step ladder, which represents the structure of society as a visual analog scale. The respondents were asked to mark where they would place themselves on this “social ladder”. The question was: “Please imagine that this ladder represents the structure of society in Germany. At the top are the people with the most money, the highest education and the best jobs. At the bottom are the people with the least money, the lowest education and the worst jobs, or without a job. Now think of your family. What do you think what rung would your family be on? Please tick a circle next to the ladder” [21].

#### Mediator variables

For individuals up to 18 years of age, KiGGS Wave 2 contained data on different dimensions of family life, particularly focusing on the parents, such as family cohesion, parental well-being, and parental stress, as well as the parenting styles and health behavior of parents, which operated as mediator variables in our analysis.

**Family cohesion** was measured with a subscale of the family climate scale developed by Schneewind et al. [60]. The values of the nine items with a response range from 1 (“disagree”) to 4 (“agree”) rated by adolescents were added and transformed into a scale from 1 to 100. Higher scores indicate better family cohesion.

For measuring **parental well-being** the “Personal Wellbeing Index (PWI) Scale” [61] was used. The PWI scale contains seven items regarding the respondents’ satisfaction, with a response scale from 0 (“no satisfaction at all”) to 10 (“completely satisfied”). Each item corresponds to one of the following quality of life domains: standard of living, health, achievements in life, relationships, safety, community-connectedness, and future security. For the index, all items were added and transformed into a scale from 1 to 100. Higher scores indicate better well-being [61].

Parental stress was operationalized by the “**number of everyday stressors**”. We examined 13 stressors by asking parents whether they felt burdened by household work, financial worries, sole responsibility for parenting, family members in need of care, parenting problems or conflicts, conflicts with an (ex-)partner or other family members, loneliness, the occupational situation or unemployment, lack of recognition of household and family chores, a disabled or chronically ill child or conflicts of compatibility of family and work (“very strong/strong” versus “slightly/not at all”) [45,62]. The additive index of the dichotomous items exhibited a range of 0–13, with higher values indicating higher levels of stress.

The **parenting behaviors** of the mother and the father were assessed using the current German version of the “Zurich short questionnaire on parenting behavior” (D-ZKE) [63]. The four parenting styles distinguished for mothers and fathers were: “authoritative” (characterized by parental warmth, support and clear rules), “emotional distancing” (characterized by low parental warmth and support), “demanding-controlling” (characterized by high parental psychological pressure, regulation and control) and “permissive” (characterized by low parental psychological pressure, rules and control). For single parent-families, the category “no mother/no father in the household” was included for the parent not living together with the adolescent [44].

Regarding present parental health and health behavior, three indicators were used. First, **parental obesity** of at least one parent (“yes”/ “no”) was measured on the basis of information about the height and weight of the mother and father reported by the interviewed parent. Parents with a body mass index of 30 kg/m^2^ or more were considered obese. Second, **parental sporting activity** was measured by asking whether at least one parent had participated in a sport in the last 3 months (regardless of the extent) (“yes”/“no”). Third, **parental smoking** was examined by asking whether at least one parent smoked daily or occasionally (“yes”). “No” indicated that neither parent smoked.

#### Control variables

**Age**, grouped into “11–13 years” (early adolescence) and “14–17 years” (middle adolescence) [64], and **migration background** (“none”, “one-sided”, “two-sided”) [65] were included as control variables. All analyses were stratified by **gender**.

### Statistical analysis

We analyzed the effects of SEP on SRH of 11–17-year-olds using linear regression models separately for income, education, occupational status, SEP index, and adolescents’ SSS. Even though SRH deviates from a normal distribution and is skewed toward higher values, linear regression models are valid and suitable for analyzing not-normally distributed outcomes in large samples [66]. To decompose the effects of SEP indicators on SRH into the direct effects of these predictors and the indirect effects through psychosocial and health behavior-related familial determinants, the KHB (Karlson-Holm-Breen) method was used [67]. This method allows the degree to which covariates (familial determinants) mediate the association between a predictor (SEP) and an outcome variable (SRH) to be quantified. In the first step of the mediation analyses, we calculated the SEP effects of the reduced models, including only one SEP indicator (total effect). In the full models, the total effect of the SEP indicator was divided into direct and indirect effects. Additionally, the percentage reduction in SEP effects due to mediation [67] for all five SEP indicators was calculated. This percentage reduction is referred to below as the “mediation percentage.” Kohler et al. [67] refer to this measure as the “confounding percentage.” For each SEP indicator, separate regression models were estimated. To enable better comparison of the coefficients, all SEP variables were z-standardized.

In a further exploratory step, the percentage contribution of each mediator variable to the indirect SEP effect is reported. For reasons of clarity, only the regression results of the SES index, which summarizes all three SEP indicators, as well as the SSS, were reported.

As a prerequisite for the mediation analysis, we analyzed whether the included familial determinants were associated with SRH as well as each SEP indicator. This was achieved using bivariate linear regression (see S1 Table in the Appendix). All familial determinants showed a significant association with each of the SEP indicators as well as SRH in at least one gender. Only family cohesion was not associated with parental education level in either female or male adolescents. The basic prerequisites for mediation were thus satisfied. However, to explore gender differences in the results of the mediation analysis, all familial determinants were included in the regressions throughout.

Finally, to analyze which socioeconomic, psychosocial or behavior-related familial determinants predicted SRH best, we conducted a linear regression considering the SEP index, the SSS, and all mediator and control variables at once. For better comparability, we also reported z-standardized beta coefficients.

Age and migration background were used as control variables in all regressions. All analyses were calculated stratified by gender. Because a cohort sample was analyzed, weighting factors were used in the statistical analyses to compensate for possible biases in the sample due to selective re-participation [68,69]. Additionally, the weighting factor adjusted the distribution of the sample to the German population regarding age, sex, region, nationality, and level of parental education.

The analyses were carried out using Stata (version 15.1 SE) software. Differences were considered statistically significant when p-values were lower than 0.05.

## Results

### Total, direct and indirect effects of the SEP indicators on SRH

Table 2 shows the associations between all SEP indicators and SRH (total effects) and the results of the decomposition of these total effects into direct and indirect effects by including familial determinants into the models.

**Table 2:** Decomposition of the total effects of SEP indicators on self-rated health into direct and indirect effects by familial determinants in female and male adolescents (linear regression, KHB method).

	Female adolescents (n = 1,838)				Male adolescents (n = 1,718)			
	Beta	95% CI		p	Beta	95% CI		p
**Income**								
Total effect	**0.057**	**0.020**	**0.094**	**0.003**	0.027	−0.012	0.066	0.181
Direct effect	0.009	−0.029	0.046	0.649	−0.005	−0.045	0.035	0.799
Indirect effect	**0.048**	**0.023**	**0.073**	**< 0.001**	**0.032**	**0.012**	**0.052**	**0.002**
R^2^ (full model)	0.126				0.087			
Mediation %	84.6%				119.3%			
**Education**								
Total effect	**0.047**	**0.007**	**0.087**	**0.022**	**0.045**	**0.005**	**0.084**	**0.027**
Direct effect	0.010	−0.030	0.050	0.619	0.016	−0.024	0.056	0.446
Indirect effect	**0.037**	**0.015**	**0.058**	**0.001**	**0.029**	**0.009**	**0.049**	**0.004**
R^2^ (full model)	0.126				0.087			
Mediation %	78.3%				65.1%			
**Occupation**								
Total effect	**0.053**	**0.012**	**0.093**	**0.011**	**0.042**	**0.003**	**0.081**	**0.036**
Direct effect	0.013	−0.029	0.056	0.537	0.026	−0.014	0.066	0.202
Indirect effect	**0.039**	**0.021**	**0.058**	**< 0.001**	0.016	−0.003	0.034	0.098
R^2^ (full model)	0.126				0.088			
Mediation %	74.7%				37.7%			
**SEP index**								
Total effect	**0.064**	**0.025**	**0.103**	**0.001**	**0.046**	**0.005**	**0.087**	**0.028**
Direct effect	0.014	−0.027	0.054	0.513	0.014	−0.027	0.056	0.501
Indirect effect	**0.051**	**0.026**	**0.075**	**< 0.001**	**0.032**	**0.009**	**0.054**	**0.005**
R^2^ (full model)	0.126				0.087			
Mediation %	78.8%				68.9%			
**SSS**								
Total effect	**0.086**	**0.043**	**0.128**	**< 0.001**	**0.097**	**0.056**	**0.138**	**< 0.001**
Direct effect	0.024	−0.014	0.062	0.221	**0.055**	**0.009**	**0.100**	**0.019**
Indirect effect	**0.062**	**0.039**	**0.085**	**< 0.001**	**0.042**	**0.021**	**0.063**	**< 0.001**
R^2^ (full model)	0.127				0.092			
Mediation %	72.3%				43.6%			

We found significant total effects on SRH for income, education, occupational status, SEP index, and SSS, but not for household income in male adolescents (research question 1).

After including familial determinants in the models (research question 2), a coherent pattern was found in female adolescents: There was no significant direct effect for any SEP indicator. Instead, the association between all SEP indicators and SRH was significantly mediated by the familial determinants. The mediation percentages indicated the proportion to which the familial determinants explained the association between each SEP indicator and SRH. More than 70% of the total effect of each SEP indicator was explained by the included familial determinants. In terms of household income, the mediation percentage was up to 84.6% in female adolescents.

In male adolescents, mediation through familial determinants was observed for income, parental education, the SEP index and the SSS, because the indirect effects for these indicators were significant. However, the mediation percentages in male adolescents for education and SEP index were below 70%, and that for SSS was only 43.6%. Regarding income, there was a confounding percentage of 119.3%, indicating that the indirect effect of income was stronger than the total effect. When the familial determinants were included in the model, the direct effect of income became negative (although this effect was not significant). Without including familial determinants as mediator variables, the direct and indirect effects of income were added and eliminated each other. A significant direct effect was only found for the SSS in male adolescents.

### Familial determinants mediating the association between SEP and SRH

In the next step, we explored which familial determinants explain the association of SRH with the SEP index or the SSS in female and male adolescents (research question 3). Table 3 shows the proportions of the differences between the full models (inclusive familial determinants) and the reduced models (exclusive familial determinants) that were contributed by each mediator variable.

**Table 3:** Proportions (in %) of the indirect effect of the familial determinants in explaining the association of the SEP index and the SSS with SRH in female and male adolescents.

	Female adolescents		Male adolescents	
Mediators	SEP index	SSS	SEP index	SSS
Family cohesion	**20.1**	**26.8**	**24.1**	**54.4**
Parental well-being	**54.3**	**45.4**	3.6	0.1
Number of stressors	−5.6	−6.2	0.2	-1.9
Parenting style mother				
Authoritative	ref		ref	
Emotional distancing	1.7	2.6	5.0	6.2
Demanding controlling	1.6	2.5	8.0	7.5
Permissive	1.7	1.3	−5.1	-1.7
No mother in household	−1.0	1.1	−0.2	0.5
Parenting style father				
Authoritative	ref	ref	ref	ref
Emotional distancing	6.1	5.5	0.4	0.5
Demanding controlling	2.3	3.4	0.2	0.1
Permissive	1.6	1.8	0.4	0.1
No father in household	**10.5**	**11.8**	**20.3**	**16.7**
Parental obesity	4.5	1.7	**22.1**	7.5
Parental sporting activity	**17.6**	7.4	−0.7	−1.3
Parental smoking	−**15.4**	−5.1	**21.7**	**11.3**

Proportions > 10% are printed in bold.

In female adolescents, parental well-being was the most important mediator. A total of 54.3% of the indirect effect of the SEP index on SRH and 45.4% of the indirect effect of the SSS on SRH could be explained by parental well-being. The proportion of family cohesion for the indirect effect of the SEP index was 20.1% and that for the SSS was 26.8%. Living in a single-mother family mediated the effect on SEP, as measured by both the SEP index (10.5%) and SSS (11.8%). Furthermore, 17.6% of the indirect effect of the SEP index on SRH was explained by parental sporting activity. Parental smoking did not explain the effect of the SEP index on SRH in females, but reduced the indirect effect through familial determinants by 15.4%, and thus reinforced the direct effect of the SEP index.

In male adolescents, family cohesion was also an important mediator, particularly for SSS, with a proportion of 54.4%. In contrast to the results for female adolescents, parental well-being was not an important mediator in male adolescents. Similar to female adolescents, we also observed a mediating effect of living in a single-mother family in male adolescents (SEP index 20.3%, SSS 16.7%). Additionally, parental smoking mediated the association between each of the SEP index (21.7%) and the SSS (11.3%) with SRH among male adolescents. Parental obesity was another mediator for the SEP index (22.1%) in male adolescents.

### Socioeconomic, psychosocial and health-behavioral determinants of SRH

Table 4 reports the full models considering SEP index as well as SSS and all familial determinants (research question 4). Not all mediators that explained the effect of the SEP index or the SSS had a significant coefficient in the full models. This was the case, for example, for single-mother-families or parental smoking. In the full models, these determinants were not significantly associated with SRH. Instead, in female adolescents, an emotionally distant parenting style of the father was associated with a poorer SRH than an authoritative parenting style, which is characterized by emotional warmth and clear rules by the father. However, the parenting style of the father was not a significant mediator and did not explain the association of the SEP index or SSS with SRH.

**Table 4:** Coefficients for SRH in female and male adolescents (linear regressions, full model with SEP index and SSS).

	Female adolescents				Male adolescents			
	Coef.	Std. err.	p	Beta	Coef.	Std. err.	p	Beta
SEP index	0.002	0.006	0.759	0.011	−0.001	0.005	0.919	−0.003
SSS	0.017	0.016	0.303	0.033	**0.043**	**0.019**	**0.023**	**0.085**
Family cohesion	**0.005**	**0.001**	**< 0.001**	**0.135**	**0.008**	**0.002**	**< 0.001**	**0.187**
Parental well-being	**0.006**	**0.002**	**< 0.001**	**0.135**	0.000	0.002	0.988	0.001
Number of stressors	0.011	0.013	0.376	0.032	0.002	0.011	0.841	0.006
Parenting style mother								
Authoritative	ref				ref			
Emotional distancing	−0.049	0.070	0.485	−0.027	−0.096	0.068	0.157	−0.056
Demanding controlling	−0.091	0.074	0.218	−0.050	−0.121	0.065	0.062	−0.072
Permissive	0.055	0.057	0.334	0.038	−0.040	0.059	0.494	−0.026
No mother in household	0.174	0.165	0.293	0.025	−0.111	0.217	0.608	−0.012
Parenting style father								
Authoritative	ref				ref			
Emotional distancing	**−0.202**	**0.075**	**0.007**	**−0.099**	−0.023	0.086	0.787	−0.011
Demanding controlling	−0.095	0.068	0.161	−0.057	−0.016	0.064	0.804	−0.010
Permissive	0.024	0.055	0.665	0.016	0.002	0.063	0.974	0.001
No father in household	−0.109	0.092	0.237	−0.052	−0.116	0.074	0.117	−0.056
Parental obesity	−0.026	0.048	0.582	−0.018	**−0.123**	**0.044**	**0.005**	**−0.084**
Parental sporting activity	0.119	0.066	0.073	0.070	−0.007	0.056	0.896	−0.004
Parental smoking	0.063	0.042	0.133	0.046	−0.050	0.042	0.236	−0.038
Age								
11−13 years	ref				ref			
14−17 years	**−0.132**	**0.044**	**0.003**	**−0.100**	−0.036	0.042	0.397	−0.027
Migration background								
No	ref				ref			
One-sided	0.053	0.062	0.390	0.024	0.056	0.064	0.381	0.026
Two-sided	−0.017	0.077	0.826	−0.009	−0.014	0.072	0.844	−0.008
Constant	2.260	0.182	0.000		2.621	0.214	0.000	
R^2^	0.127				0.092			

In female adolescents, good family cohesion and high parental well-being were the strongest predictors of self-rated good health. Furthermore, a significant effect of age was seen in female adolescents, with older females rating their health as worse than younger females. All predictor variables together explained 12.7% of the variance of SRH in females (R^2^).

In male adolescents, good family cohesion was the strongest predictor of self-rated good health. With the focus on the SEP indicators, the effect of male adolescents’ SSS on SRH remained significant, independent of other predictors. This finding reflects that a high ranking on the social ladder was associated with better general health. In contrast, parental obesity was associated with worse general health in males. No other predictors reached statistical significance and were therefore not independently associated with SRH. However, it should be noted that, in male adolescents, the parenting style of the mother was associated with SRH, whereas, in females, the parenting style of the father was associated with SRH. Compared with the authoritative parenting style, male adolescents with a mother who exhibited a demanding controlling parenting style rated their health as worse. However, the effect was not significant at the 95% level. The R^2^ value for the male adolescents’ model was 0.092, indicating that 9.2% of the variance of SRH was explained by all predictors together.

## Discussion

The aim of the current study was to analyze the associations between different SEP indicators (household income, parental education level, parental occupational status, SEP index and SSS) and adolescents’ SRH, as well as possible mediation effects through several familial determinants.

Regarding research question 1, we found significant total effects for all SEP indicators on both female and male adolescents’ SRH, with the exception of income in male adolescents. These results were in accord with previous studies reporting significant associations between different SEP indicators and SRH [14,21,49]. However, the absence of a significant association between income and SRH in male adolescents differed from the results of the KiGGS Wave 2 cross-sectional study, in which a significant income gradient among both girls and boys aged 3–17 years was found for parent-rated health [21,70]. The comparability of these findings is limited because of differences in the included age groups (11–17 years versus 3–17 years), outcome measurements (self- versus parent-rated health), analysis strategies (metric versus categorical variables), and sample sizes [70].

Overall, the results regarding gender differences in the associations between SEP and SRH were highly heterogeneous and partially depended on the examined SEP indicator. Salonna et al. [22] reported that, among male adolescents, parental educational level, family affluence and financial strain were associated with SRH, whereas, among female adolescents, only family affluence was associated with SRH. In another study, Salonna et al. [23] reported that the socioeconomic differences in SRH in male adolescents were minor and much smaller than those in female adolescents. However, the researchers in that study investigated parental educational level and occupational status but not the families’ income situation [23]. In addition, Jeon et al. [71] observed a significant association between parental educational level and SRH in female adolescents, but not in male adolescents.

When familial determinants were considered in the current analysis (research question 2), there were no direct effects of parental SEP for household income, parental education level, parental occupational status or SEP index on female and male adolescents’ SRH. Rather, the association between parental SEP and SRH could be explained to a large extent by familial determinants. This pattern was particularly noticeable among female adolescents. Important family mediators (research question 3) included family cohesion and parental well-being, but also health behavior-related determinants such as parental smoking, parental sporting activity and obesity in at least one parent. In male adolescents, however, the SSS had a significant direct effect on SRH. This indicates that the included familial determinants could not explain the association between SSS and SRH in total. Even if the SES index was included, the SSS remained a significant predictor of SRH regardless of the parents’ SEP.

The current results share a number of similarities with existing research findings, and extend current knowledge in several ways. First, the current findings suggested that psychosocial and health-behavioral familial determinants were closely associated with families’ SEP, and that SEP in adolescence has an indirect effect through family life rather than a direct effect on adolescents’ health. This phenomenon was previously reported by Sweeting and West [72] who found that family life may have stronger direct effects on health than material factors in adolescence.

Specifically, the current results revealed that poor family cohesion, low parental well-being and poor health-related behavior by parents were the mechanisms by which low SEP resulted in worse health of adolescents. However, the number of everyday stressors and maternal and paternal parenting style were not significant mediators in the current study. It could be assumed that parenting style is strongly correlated with family cohesion. Similar to parenting behavior, family cohesion also covers aspects of parental warmth and control as well as shared leisure time in the family [63]. Regarding everyday stressors (e.g., compatibility of family life and work or parenting problems), it is possible that the type of stressor has a stronger influence than the number of stressors [45]. Several previous studies also reported that psychosocial familial determinants mediated the association between parental SEP and adolescents’ SRH [19,22,49]. This was the case for family cohesion [19], parental support [22] and the relationship with the father [49]. Overall, the current results can be interpreted in light of the family stress model [33], although we did not use the same familial determinants. According to the family stress model, financial hardship and economic pressure lead to parental stress and, as a result, less favorable parenting and a poorer parent-child relationship, which, in turn, leads to worse health in young people. Regarding parental well-being, we observed a mediating effect on social disparities in adolescents’ SRH. Bøe et al. [73] also identified maternal and paternal emotional well-being as mediators for the association of family economic status and maternal education with externalizing and internalizing problems in young adolescents. In addition, we found that the health behavior of parents also plays a mediating role in health inequality in adolescence. Thus, the stress of parents due to social deprivation and low social resources appears to lead to unhealthy behavior or obesity in parents, and this, in turn, affects the health of adolescents. However, Bauldrey et al. [40] found no evidence for this mechanism, reporting that parental obesity and smoking in the household did not mediate the association between childhood SEP and SRH in adolescence and young adulthood.

Second, regarding the hypothesis of equalization in health of youth [24,25], our results revealed that the SEP of parents is relevant to health in adolescence, but that its effect largely occurs through family cohesion, family practices and parental health-behavior, as well as parental well-being. This finding may be helpful for specifying the mechanisms that lead to health inequalities among adolescents. However, further research is required to elucidate the mediation effects of psychosocial and health behavioral familial determinants in childhood [74] and young adulthood.

Third, adolescents’ SSS appeared to play an important role in subjective health in the current study, particularly in males. This finding is in line with the results of previous studies [18,71]. The result highlights that SSS, rather than objective measures of family SEP, is associated with adolescents’ health [21,71].

Fourth, the mediation of health inequalities by familial determinants appeared to be stronger among female than male adolescents in the current study. This finding indicates that the family plays a more influential role among female than among male adolescents. Sweeting and West [72] found that in female adolescents, family conflict and the quality of relationships with parents were more consistently related to self-esteem, mental well-being and physical symptoms, compared with male adolescents. Similarly, Lin et al. [75] reported that female adolescents were more sensitive to family environment factors (e.g., family arguments, quarrels with parents) than male adolescents with regard to psychological well-being. Wang et al. [76] observed that regarding non-medical use of prescription drugs female adolescents were more sensitive to the communication problems with the mother and more easily affected by parental behaviors such as problematic drinking compared with male adolescents. In reference to Gilligan’s theory of female development [77], Ohannessian [78] argued that the development of the self during adolescence is more closely related to attachment and relationships with others among females, compared with males. Accordingly, female adolescents are more relationship-oriented than male adolescents, for whom emotional separation from others seems to be more important [78]. This approach may provide an explanation for the gender differences we observed in the current study. However, this assumption requires further research. Another potential explanation is that SRH is defined differently by female and male adolescents. In Germany, significant gender differences in the extent to which SRH is associated with adolescents’ mental health have previously been identified [13]. Specifically, the association between mental health and SRH was reported to be greater in female than in male adolescents, whereas no gender differences were observed in the association between physical health and SRH [13]. Regarding the family stress model [33], there is substantial evidence that in particular inequalities in mental health can be explained by familial determinants.

### Limitations and strengths

An important limitation of the current analysis is related to the cohort study design. Although we used a weighting factor to account for unequal sampling probabilities and to adjust the distribution of the sample to the demographics of the German population, the drop out weighting factor did not provide the same degree of representativeness as a cross-sectional study based on a random sample.

Another limitation of our study is that it relied on cross-sectional data because some familial determinants were surveyed for the first time in KiGGS Wave 2. Therefore, a causal mechanism can be assumed but is not substantiated by our analysis, and confirmation would require longitudinal data. Alternatively, selection processes may partially explain the associations by which poor parental well-being is caused by a parent’s chronic disease, resulting in a lower SEP. In addition, poor health of adolescents may influence family cohesion, parental well-being or the time available for parents to engage in sporting activities. Socioeconomic, psychosocial and behavior-related determinants and health, however, are closely interwoven throughout the entire course of life and influence one another, which can even present challenges for longitudinal analysis.

Another limitation of the current study is the focus on parental determinants. Adolescents’ relationships with siblings (including conflict and mutual support) and the roles of other relatives or people in the wider family environment (e.g., grandparents) who often take on important care tasks were not considered in this analysis. However, because we analyzed inequalities based on income, parental educational level and parental occupational status, we consider that the focus on parents was justified and appropriate.

Importantly, only answers from one parent were considered. For some determinants the parental participant also provided proxy information about the other parent. This was the case for smoking, height and weight, and sporting activities, but also for educational level and occupational status. Some previous studies have reported that the impacts of mothers and fathers on adolescents’ health can be different [49,79]. Regarding the explanation of the inequalities in SRH by familial determinants, the relationship with the father [49] as well as the father’s support [23] were found to be particularly important. In future studies, it may be helpful to consider information from both parents separately, as was performed in the current study for parenting style. However, this approach is difficult with single-parent families because missing values for the parent not living in the household must be considered.

In this analysis, the family structure is only considered indirectly. However, in future studies the role of family structure should be analyzed more in-depth because the family structure could be a mediator. Thus, the family stress model [33] assumes that family economic pressure leads to parental conflict and, as a possible result, to the separation of the parents, consequently impacting the health of adolescents. However, there is also empirical evidence that living in a single-parent family is associated with lower SEP and potentially with poverty, which then results in poorer adolescent health [80]. Again, longitudinal analyses will be required to determine which effect is stronger.

A familial determinant not considered in our analysis was the parents’ employment status [81–83]. Similar to the family structure, employment status cannot be unequivocally classified as a mediator in the association between parental SEP and adolescents’ SRH, because, on the one hand, parents’ educational level affects their labor market attachment, while, on the other hand, parents’ employment status has a strong influence on family income. Therefore, further studies that analyze the different directions of association would be useful.

Another difficulty is the interweaving of the various socioeconomic, psychosocial and health-behavioral variables included in our analysis. This was particularly evident when income, educational level and occupational status were included concurrently in one model. Therefore, we used the SEP index, which comprises the combined effects of income, education and occupation in an additive score.

The strengths of our analysis included a relatively large sample size and the ability to integrate several SEP indicators as well as several familial determinants. Another strength is that we included information from the adolescents themselves as well as from parents. Young people are often unable to provide precise information about their parents’ income, educational level, occupational status, or well-being. Additionally, when only information from parents is considered, it has been reported that the well-being of the parent can impact the assessment of other variables, including adolescents’ health [84]. Overall, the current findings can make a contribution toward explaining health inequalities in adolescence.

### Conclusions

The current analysis verified associations between families’ SEP and adolescents’ SRH. The mediation analysis also revealed that socioeconomic disadvantage can lead to poorer health in adolescents through a lack of family cohesion or family practices in everyday life. Further longitudinal studies are necessary to analyze the mechanisms of “doing family” [7] in more detail. Additionally, the social contexts around the adolescents and the family, such as school, vocational training, sports and leisure facilities, should also be considered. However, because the family acts as the central interface to other institutional contexts it influences many factors, such as the academic achievement of young people [10]. Investigating whether the current findings also apply to younger children (pre-school and primary school age) and young adults may also produce valuable insights.

The current study is embedded in a larger research unit that analyzes the roles of different institutional contexts, including the family, kindergarten, primary and secondary school, vocational training, university, labor market and health care system, in explaining health inequalities among young people [85].

Several conclusions can be drawn from the current findings. Reducing health inequalities in adolescence requires policy interventions on the macro-level, community-based strategies on the meso-level and programs to improve the parenting competence of parents and family functioning on the micro-level. This includes policies that aim to reduce poverty in families. In addition, activities are needed to better integrate non-employed parents into the labor market, which could improve parents’ financial situation as well as their psychosocial well-being, and may also contribute to a clearly structured daily routine in families. Furthermore, targeted school support and better educational opportunities are needed, especially for adolescents from socially disadvantaged families. However, social disadvantage does not only refer to the socioeconomic situation, but also to psychosocial and behavioral factors that are mutually interwoven. Interventions that aim to strengthen family cohesion or parenting skills in high conflict-families with low social resources may be valuable. In addition, interventions that lead to an improvement in parents’ well-being may also be helpful for promoting the well-being and health of adolescents. Furthermore, the provision of appropriate infrastructure (e.g., sufficient green areas in neighborhoods) as well as low-threshold services (e.g., from sports clubs) should be considered, which can promote healthy behavior among parents and young people. Even more compensatory-oriented services for adolescents outside the family (such as healthy lunches at school) could enable adolescents to lead healthier lives, and could relieve stress for their parents. Finally, evaluation studies are needed to investigate which health promotion interventions are able to most effectively reach socially disadvantaged young people and their families.

## Supporting information

S1 Table P-values for associations between SRH and SEP indicators with familial determinants (results of bivariate linear regression analyses). *** p < 0.001 ** p < 0.01 * p < 0.05 n.s. not significant.
(DOCX)

## References

[1] Kramer MR, Schneider EB, Kane JB, Margerison-Zilko C, Jones-Smith J, King K, et al. Getting under the skin: children’s health disparities as embodiment of social class. Popul Res Policy Rev. 2017;36(5):671–97. doi: 10.1007/s11113-017-9431-7 29398742 PMC5791911

[2] Viner RM, Ozer EM, Denny S, Marmot M, Resnick M, Fatusi A, et al. Adolescence and the social determinants of health. Lancet. 2012;379(9826):1641–52. doi: 10.1016/S0140-6736(12)60149-4 22538179

[3] Lampert T, Hoebel J, Kuntz B, Finger JD, Hölling H, Lange M, et al. Health inequalities among children and adolescents in Germany. Developments over time and trends from the KiGGS study. Journal of Health Monitoring. 2019;4(1):15–37. Available from: https://edoc.rki.de/bitstream/handle/176904/5912/JoHM_01_2019_Health_Inequalities.pdf?sequence=1&isAllowed=y. doi: 10.25646/5871 35146241 PMC8822245

[4] Hagell A, Shah R, Viner R, Hargreaves D, Varnes L, Heys M. The social determinants of young people’s health. Identifying the key issues and assessing how young people are doing in the 2010s. Health Foundation working paper. AYPH Working Paper 01. London: Health Foundation; 2018. Available from: https://www.health.org.uk/sites/default/files/The-social-determinants-of%20-young-peoples-health_0.pdf.

[5] Kolip P, Lademann J. [Family and health] Familie und Gesundheit. In: Hurrelmann K, Razum O, editors. [Handbook of health sciences] Handbuch Gesundheitswissenschaften. Weinheim: Beltz Juventa; 2012. p. 517–40. German.

[6] Federal Ministry for Family Affairs, Senior Citizens, Women and Youth (Germany), editor. [12. Children and Youth Report. Report on the living situation of young people and the services of child and youth welfare in Germany] 12. Kinder- und Jugendbericht. Bericht über die Lebenssituation junger Menschen und die Leistungen der Kinder- und Jugendhilfe in Deutschland. Berlin: Federal Ministry for Family Affairs, Senior Citizens, Women and Youth; 2005. German. Available from: https://www.bmfsfj.de/resource/blob/112224/7376e6055bbcaf822ec30fc6ff72b287/12-kinder-und-jugendbericht-data.pdf.

[7] Jurczyk K, Lange A, Thiessen B, editors. [Doing family. Why family life can no longer be taken for granted today] Doing Family. Warum Familienleben heute nicht mehr selbstverständlich ist. Weinheim, Basel: Beltz Juventa; 2014. German.

[8] Havighurst RJ. Developmental tasks and education. New York: David McKay; 1948.

[9] Federal Ministry for Family Affairs, Senior Citizens, Women and Youth (Germany), editor. [14. Children and Youth Report. Report on the living situation of young people and the services of child and youth welfare in Germany. Report of the Expert Commission] 14. Kinder- und Jugendbericht. Bericht über die Lebenssituation junger Menschen und die Leistungen der Kinder- und Jugendhilfe in Deutschland. Bericht der Sachverständigenkommission. Berlin: Federal Ministry for Family Affairs, Senior Citizens, Women and Youth; 2013. German. Available from: https://www.bmfsfj.de/resource/blob/93146/6358c96a697b0c3527195677c61976cd/14-kinder-und-jugendbericht-data.pdf.

[10] Walper S, Gniewosz B. [The importance of the family in adolescence] Die Bedeutung der Familie im Jugendalter. In: Gniewosz B, Titzmann PF, editors. [Handbook Youth. Psychological perspectives on changes in adolescence] Handbuch Jugend. Psychologische Sichtweisen auf Veränderungen in der Adoleszenz. Stuttgart: W. Kohlhammer; 2018. p. 71–88. German.

[11] Fosse NE, Haas SA. Validity and stability of self-reported health among adolescents in a longitudinal, nationally representative survey. Pediatr. 2009;123(3):e496–501. 10.1542/peds.2008-1552 19254984

[12] EUROSTAT, editor. European Health Interview Survey (EHIS wave 3). Methodological manual. Re-edition. Luxembourg: Publications Office of the European Union; 2020. Available from: https://ec.europa.eu/eurostat/documents/3859598/10820524/KS-01-20-253-EN-N.pdf/2d66d5d7-b966-38ba-881a-a8f4b6d3f5e0?t=1588680461000.

[13] Baćak V, Ólafsdóttir S. Gender and validity of self-rated health in nineteen European countries. Scand J Public Health. 2017;45(6):647–53. doi: 10.1177/1403494817717405 28673121

[14] Lampert T, Hagen C, Heizmann B. [Health inequalities in children and adolescents in Germany] Gesundheitliche Ungleichheit bei Kindern und Jugendlichen in Deutschland. Robert Koch-Institut; 2010. p. 86. German.

[15] Lampert T, Kuntz B, KiGGS Study Group. [Growing up healthy–What importance does social status have?] Gesund aufwachsen–Welche Bedeutung kommt dem sozialen Status zu? GBE kompakt. 2015;6(1). German. Available from: https://www.rki.de/DE/Content/Gesundheitsmonitoring/Gesundheitsberichterstattung/GBEDownloadsK/2015_1_gesund_aufwachsen.pdf?__blob=publicationFile.

[16] Richter M, Moor I, van Lenthe FJ. Explaining socioeconomic differences in adolescent self-rated health: the contribution of material, psychosocial and behavioural factors. J Epidemiol Community Health. 2012;66(8):691–7. doi: 10.1136/jech.2010.125500 21543387

[17] Spencer NJ. Social equalization in youth: evidence from a cross-sectional British survey. Eur J Public Health. 2006;16(4):368–75. doi: 10.1093/eurpub/cki222 16431870

[18] Plenty S, Mood C. Money, Peers and parents: social and economic aspects of inequality in youth wellbeing. J Youth Adolesc. 2016;45(7):1294–308. doi: 10.1007/s10964-016-0430-5 26847325 PMC4901095

[19] Rattay P, Lampert T, Neuhauser H, Ellert U. [Significance of family life for the health of children and adolescents. Results of the German Health Interview and Examination Survey for Children and Adolescents (KiGGS)] Bedeutung der familialen Lebenswelt für die Gesundheit von Kindern und Jugendlichen. Ergebnisse des Kinder- und Jugendgesundheitssurveys (KiGGS). Z Erziehungswiss. 2012;15(1):145–70. German.

[20] Torsheim T, Nygren JM, Rasmussen M, Arnarsson AM, Bendtsen P, Schnohr CW, et al. Social inequalities in self-rated health: a comparative cross-national study among 32,560 Nordic adolescents. Scand J Public Health. 2018;46(1):150–6. doi: 10.1177/1403494817734733 29039236

[21] Lampert T, Hoebel J, Kuntz B, Müters S, Kroll LE. Socioeconomic status and subjective social status measurement in KiGGS Wave 2. Journal of Health Monitoring. 2018;3(1). Available from: https://edoc.rki.de/bitstream/handle/176904/5639/JoHM_01_2018_Socioeconomic_Status_KiGGS-Wave2.pdf?sequence=1&isAllowed=y. 10.17886/RKI-GBE-2018-033 PMC8848848 35586179

[22] Salonna F, Geckova AM, Zezula I, Sleskova M, Groothoff JW, Reijneveld SA, et al. Does social support mediate or moderate socioeconomic differences in self-rated health among adolescents? Int J Public Health. 2012;57(3):609–17. doi: 10.1007/s00038-011-0300-6 21912942 PMC3359452

[23] Salonna F, van Dijk JP, Geckova AM, Bacikova-Sleskova M, Groothoff JW, Reijneveld SA. Changes in socio-economic differences in adolescent self-reported health between 15 and 19 years of age: a longitudinal study. Public Health. 2014;128(4):380–3. doi: 10.1016/j.puhe.2013.11.009 24461261

[24] West P. Health inequalities in the early years: is there equalisation in youth? Soc Sci Med. 1997;44(6):833–58. doi: 10.1016/s0277-9536(96)00188-8 9080566

[25] West P, Sweeting H. Evidence on equalisation in health in youth from the West of Scotland. Soc Sci Med. 2004;59(1):13–27. doi: 10.1016/j.socscimed.2003.12.004 15087139

[26] Green MA. The equalisation hypothesis and changes in geographical inequalities of age based mortality in England, 2002–2004 to 2008–2010. Soc Sci Med. 2013;87:93–8. doi: 10.1016/j.socscimed.2013.03.029 23631783

[27] Chen E, Matthews KA, Boyce WT. Socioeconomic differences in children’s health: how and why do these relationships change with age? Psychol Bull. 2002;128(2):295–329. doi: 10.1037/0033-2909.128.2.295 11931521

[28] Chen E, Martin AD, Matthews KA. Socioeconomic status and health: do gradients differ within childhood and adolescence? Soc Sci Med. 2006;62(9):2161–70. doi: 10.1016/j.socscimed.2005.08.054 16213644

[29] Murasko JE. An evaluation of the age-profile in the relationship between household income and the health of children in the United States. J Health Econ. 2008;27(6):1489–502. doi: 10.1016/j.jhealeco.2008.07.012 18774615

[30] Quon EC, McGrath JJ. Subjective socioeconomic status and adolescent health: a meta-analysis. Health Psychol. 2014;33(5):433–47. doi: 10.1037/a0033716 24245837 PMC5756083

[31] Goodman E, Huang B, Schafer-Kalkhoff T, Adler NE. Perceived socioeconomic status: a new type of identity that influences adolescents’ self-rated health. J Adolesc Health. 2007;41(5):479–87. doi: 10.1016/j.jadohealth.2007.05.020 17950168 PMC2204090

[32] Federal Statistical Office (Germany). [At-risk-of-poverty rate measured on the federal median by household type] Armutsgefährdungsquote gemessen am Bundesmedian nach Haushaltstyp. 2021. German. Available from: https://www.destatis.de/DE/Themen/Gesellschaft-Umwelt/Soziales/Sozialberichterstattung/Tabellen/06agq-zvbm-haushaltstyp.html.

[33] Conger RD, Conger KJ, Martin MJ. Socioeconomic status, family processes, and individual development. J Marriage Fam. 2010;72(3):685–704. doi: 10.1111/j.1741-3737.2010.00725.x 20676350 PMC2910915

[34] Scharte M, Bolte G. Increased health risks of children with single mothers: the impact of socio-economic and environmental factors. Eur J Public Health. 2013;23(3):469–75. doi: 10.1093/eurpub/cks062 22683774

[35] McMunn AM, Nazroo JY, Marmot MG, Boreham R, Goodman R. Children’s emotional and behavioural well-being and the family environment: findings from the Health Survey for England. Soc Sci Med. 2001;53(4):423–40. doi: 10.1016/s0277-9536(00)00346-4 11459394

[36] Meland E, Breidablik HJ, Thuen F. Family factors predicting self-rated health during early adolescence. Scand J Public Health. 2021;49(5):546–54. doi: 10.1177/1403494820972282 33245020

[37] Rattay P, von der Lippe E, Mauz E, Richter F, Hölling H, Lange C, et al. Health and health risk behaviour of adolescents—Differences according to family structure. Results of the German KiGGS cohort study. PLOS ONE. 2018;13(3):e0192968. doi: 10.1371/journal.pone.0192968 29513693 PMC5841741

[38] Breidablik HJ, Meland E, Holmen TL, Lydersen S. Role of parents in adolescent self-rated health: Norwegian Nord-Trøndelag Health Study. Adolesc Health Med Ther. 2010;1:97–104. doi: 10.2147/AHMT.S12877 24600265 PMC3915975

[39] Herke M, Knöchelmann A, Richter M. Health and well-being of adolescents in different family structures in Germany and the importance of family climate. Int J Environ Res Public Health. 2020;17(18). doi: 10.3390/ijerph17186470 32899489 PMC7559242

[40] Bauldry S, Shanahan MJ, Boardman JD, Miech RA, Macmillan R. A life course model of self-rated health through adolescence and young adulthood. Soc Sci Med. 2012;75(7):1311–20. doi: 10.1016/j.socscimed.2012.05.017 22726620 PMC4297471

[41] Conger R, Dogan S. Social class and socialization in families. In: Grusec JE, Hastings PD, editors. Handbook of socialization. Theory and research. New York: Guilford Press; 2007. p. 433–60.

[42] Conger RD, Donnellan MB. An interactionist perspective on the socioeconomic context of human development. Annu Rev Psychol. 2007;58:175–99. doi: 10.1146/annurev.psych.58.110405.085551 16903807

[43] Cobb-Clark DA, Salamanca N, Zhu A. Parenting style as an investment in human development. J Popul Econ. 2019;32(4):1315–52.

[44] Azman Ö, Mauz E, Reitzle M, Geene R, Hölling H, Rattay P. Associations between parenting style and mental health in children and adolescents aged 11–17 years: results of the KiGGS cohort study (second follow-up). Children. 2021;8(8):672. doi: 10.3390/children8080672 34438563 PMC8394813

[45] Bolster M, Rattay P, Hölling H, Lampert T. [Association between parental stress and the mental health of children and adolescents] Zusammenhang zwischen elterlichen Belastungen und der psychischen Gesundheit von Kindern und Jugendlichen. Kindheit und Entwicklung. 2020;29(1):30–9. German.

[46] Rattay P, von der Lippe E, Lampert T, KiGGS Study Group. [Health of children and adolescents in single-parent, step-, and nuclear families: results of the KiGGS study: first follow-up (KiGGS Wave 1)] Gesundheit von Kindern und Jugendlichen in Eineltern-, Stief- und Kernfamilien. Ergebnisse der KiGGS-Studie–Erste Folgebefragung (KiGGS Welle 1). Bundesgesundheitsblatt Gesundheitsforschung Gesundheitsschutz. 2014;57(7):860–8. German. doi: 10.1007/s00103-014-1988-2 24950835

[47] Blume M, Rattay P, Hoffmann S, Spallek J, Sander L, Herr R, et al. Health inequalities in children and adolescents: a scoping review of the mediating and moderating effects of family characteristics. Int J Environ Res Public Health. 2021. doi: 10.3390/ijerph18157739 34360031 PMC8345625

[48] Bacikova-Sleskova M, Benka J, Orosova O. Parental employment status and adolescents’ health: the role of financial situation, parent-adolescent relationship and adolescents’ resilience. Psychol Health. 2015;30(4):400–22. doi: 10.1080/08870446.2014.976645 25322966

[49] Moor I, Rathmann K, Stronks K, Levin K, Spallek J, Richter M. Psychosocial and behavioural factors in the explanation of socioeconomic inequalities in adolescent health: a multilevel analysis in 28 European and North American countries. J Epidemiol Community Health. 2014;68(10):912–21. doi: 10.1136/jech-2014-203933 25031452

[50] Mauz E, Lange M, Houben R, Hoffmann R, Allen J, Gößwald A, et al. Cohort profile: KiGGS cohort longitudinal study on the health of children, adolescents and young adults in Germany. Int J Epidemiol. 2019;49(2):375–k. 10.1093/ije/dyz231 PMC7266535 31794018

[51] Lange M, Hoffmann R, Mauz E, Houben R, Gößwald A, Schaffrath Rosario A, et al. KiGGS Wave 2 longitudinal component–data collection design and developments in the numbers of participants in the KiGGS cohort. Journal of Health Monitoring. 2018;3(1). Available from: https://edoc.rki.de/bitstream/handle/176904/5638/JoHM_01_2018_Longitudinal_Component_KiGGS-Wave2.pdf?sequence=1&isAllowed=y. 10.17886/RKI-GBE-2018-035 PMC8848915 35586182

[52] Federal Ministry of Labour and Social Affairs (Germany), editor. [Life situations in Germany. The German Federal Government’s 5th Report on Poverty and Wealth] Lebenslagen in Deutschland. Der 5. Armuts- und Reichtumsbericht der Bundesregierung. 2017. German. Available from: https://www.armuts-und-reichtumsbericht.de/SharedDocs/Downloads/Berichte/5-arb-langfassung.pdf?__blob=publicationFile&v=6.

[53] Lampert T, Kroll LE. [Measurement of the socioeconomic status in social epidemiological studies] Die Messung des sozioökonomischen Status in sozialepidemiologischen Studien. In: Richter M, Hurrelmann K, editors. [Health inequality: foundation, problems, perspectives] Gesundheitliche Ungleichheit: Grundlagen, Probleme, Perspektiven. Wiesbaden: VS Verlag für Sozialwissenschaften; 2009. p. 309–34. German.

[54] Lampert T, Kroll LE, Muters S, Stolzenberg H. [Measurement of the socioeconomic status within the German Health Update 2009 (GEDA)] Messung des sozioökonomischen Status in der Studie “Gesundheit in Deutschland aktuell” (GEDA). Bundesgesundheitsblatt Gesundheitsforschung Gesundheitsschutz. 2013;56(1):131–43. German. doi: 10.1007/s00103-012-1583-3 23275948

[55] Brauns H, Scherer S, Steinmann S. The CASMIN educational classification in international comparative research. In: Hoffmeyer-Zlotnik JHP, Wolf C, editors. Advances in cross-national comparison: a European working book for demographic and socio-economic variables. Boston, MA: Springer US; 2003. p. 221–44.

[56] Ganzeboom HBG, Treiman DJ. Three internationally standardised measures for comparative research on occupational status. In: Hoffmeyer-Zlotnik JHP, Wolf C, editors. Advances in cross-national comparison: a European working book for demographic and socio-economic variables. Boston, MA: Springer US; 2003. p. 159–93.

[57] International Labour Organization, editor. International Standard Classification of Occupations. ISCO-08: structure, group definitions and correspondence tables. Geneva: International Labour Organization; 2012. Available from: https://www.ilo.org/wcmsp5/groups/public/—dgreports/—dcomm/—publ/documents/publication/wcms_172572.pdf.

[58] Avvisati F. The measure of socio-economic status in PISA: a review and some suggested improvements. Large-scale Assess Edu. 2020;8(1):8.

[59] Goodman E, Adler NE, Kawachi I, Frazier AL, Huang B, Colditz GA. Adolescents’ perceptions of social status: development and evaluation of a new indicator. Pediatr. 2001;108(2):E31. doi: 10.1542/peds.108.2.e31 11483841

[60] Schneewind K, Beckmann M, Hecht-Jackl A. [Family climate scales. A report] Familienklima-Skalen. Bericht. München: Institut für Psychologie, Persönlichkeitspsychologie und Psychodiagnostik der Ludwig-Maximilians-Universität; 1985. German.

[61] International Wellbeing Group. Personal Wellbeing Index: 5th Edition. Melbourne: Australian: Centre on Quality of Life, Deakin University; 2013. Available from: http://www.acqol.com.au/instruments#measures.

[62] Sperlich S. [Psychosocial stress—an aspect of health inequality in mothers?] Psychosoziale Belastungen—ein Aspekt gesundheitlicher Ungleichheit von Müttern? In: Collatz J, editor. [Family medicine in Germany: Necessity, dilemma, perspectives] Familienmedizin in Deutschland: Notwendigkeit, Dilemma, Perspektiven. Lengerich: Pabst Science Publishers; 2010. p. 132–53. German.

[63] Reitzle M, Metzke CW, Steinhausen H-C. [Parents and children: the Zurich short-questionnaire of educational behavior (ZKE)] Eltern und Kinder: Der Zürcher Kurzfragebogen zum Erziehungsverhalten (ZKE). Diagnostica. 2001;47(4):196–207. German.

[64] Allen B, Waterman H. Stages of adolescence 2019. Available from: https://www.healthychildren.org/English/ages-stages/teen/Pages/Stages-of-Adolescence.aspx.

[65] Schenk L, Ellert U, Neuhauser H. [Children and adolescents in Germany with a migration background. Methodical aspects in the German Health Interview and Examination Survey for Children and Adolescents (KiGGS)] Kinder und Jugendliche mit Migrationshintergrund in Deutschland. Methodische Aspekte im Kinder- und Jugendgesundheitssurvey (KiGGS). Bundesgesundheitsblatt Gesundheitsforschung Gesundheitsschutz. 2007;50(5–6):590–9. German. doi: 10.1007/s00103-007-0220-z 17514443

[66] Lumley T, Diehr P, Emerson S, Chen L. The importance of the normality assumption in large public health data sets. Annu Rev Public Health 2002;23:151–69. doi: 10.1146/annurev.publhealth.23.100901.140546 11910059

[67] Kohler U, Karlson KB, Holm A. Comparing coefficients of nested nonlinear probability models. Stata Journal. 2011;11(3):420–38.

[68] Hoffmann R, Lange M, Butschalowsky H, et al. KiGGS Wave 2 cross-sectional study–participant acquisition, response rates and representativeness. Journal of Health Monitoring. 2018;3(1):82–96. Available from: https://edoc.rki.de/bitstream/handle/176904/5637/JoHM_01_2018_Cross-sectional_Study_KiGGS-Wave2.pdf?sequence=1&isAllowed=y. 10.17886/RKI-GBE-2018-032 PMC8848911 35586176

[69] Mauz E, Gößwald A, Kamtsiuris P, et al. New data for action. Data collection for KiGGS Wave 2 has been completed. Journal of Health Monitoring. 2017;2(S3):2–28. Available from: https://edoc.rki.de/bitstream/handle/176904/2812/25Pxmf2fcHqRM.pdf?sequence=1&isAllowed=y. 10.17886/RKI-GBE-2017-105 PMC10291840 37377941

[70] Lampert T, Kuntz B. [Effects of poverty for health and health behavior of children and adolescents. Results from KiGGS Wave 2] Auswirkungen von Armut auf den Gesundheitszustand und das Gesundheitsverhalten von Kindern und Jugendlichen. Bundesgesundheitsblatt Gesundheitsforschung Gesundheitsschutz. 2019;62(10):1263–74. German. doi: 10.1007/s00103-019-03009-6 31529186

[71] Jeon G-S, Ha Y, Choi E. Effects of objective and subjective socioeconomic status on self-rated health, depressive symptoms, and suicidal ideation in adolescents. Child Indic Res. 2013;6(3):479–92.

[72] Sweeting H, West P. Family life and health in adolescence: a role for culture in the health inequalities debate? Soc Sci Med. 1995;40(2):163–75. doi: 10.1016/0277-9536(94)e0051-s 7899929

[73] Bøe T, Sivertsen B, Heiervang E, Goodman R, Lundervold AJ, Hysing M. Socioeconomic status and child mental health: the role of parental emotional well-being and parenting practices. J Abnorm Child Psychol. 2014;42(5):705–15. doi: 10.1007/s10802-013-9818-9 24150864

[74] Hoffmann S, Sander L, Wachtler B, Blume M, Schneider S, Herke M, et al. Moderating or mediating effects of family characteristics on socioeconomic inequalities in child health in high‑income countries: a scoping review. BMC Public Health. 2022;22(1):338. doi: 10.1186/s12889-022-12603-4 35177014 PMC8851861

[75] Lin FG, Chou YC, Wu CH, Lin JD. Short-term and long-term influences of family arguments and gender difference on developing psychological well-being in Taiwanese adolescents. Res Dev Disabil. 2014;35(11):2735–43. doi: 10.1016/j.ridd.2014.07.018 25077832

[76] Wang W, Luo M, Xi C, Lei Y, Pan S, Gao X, et al. Cross-sectional study on influence of the family environment on the lifetime non-medical use of prescription drugs among Chinese adolescents in Guangdong: an analysis of sex differences. BMJ Open. 2019;9(7):e026758. doi: 10.1136/bmjopen-2018-026758 31278096 PMC6615848

[77] Gilligan C. In a different voice: psychological theory and women’s development. Cambridge, MA: Harvard University Press; 1983.

[78] Ohannessian CM. Parental problem drinking and adolescent psychological problems: the moderating effect of adolescent–parent communication. Youth Soc. 2013;45(1):3–26.

[79] Balaj M, York HW, Sripada K, Besnier E, Vonen HD, Aravkin A, et al. Parental education and inequalities in child mortality: a global systematic review and meta-analysis. Lancet. 2021;398(10300):608–20. doi: 10.1016/S0140-6736(21)00534-1 34119000 PMC8363948

[80] Beiser M, Hou F, Hyman I, Tousignant M. Poverty, family process, and the mental health of immigrant children in Canada. Am J Public Health. 2002;92(2):220–7. doi: 10.2105/ajph.92.2.220 11818295 PMC1447046

[81] LindholdtL, LundT, AndersenJH, LabriolaM. Labour market attachment among parents and self-rated health of their offspring: an intergenerational study. Eur J Public Health. 2020;30(3):600–5. doi: 10.1093/eurpub/ckz213 31793997

[82] Sleskova M, Salonna F, Geckova AM, Nagyova I, Stewart RE, van Dijk JP, et al. Does parental unemployment affect adolescents’ health? J Adolesc Health. 2006;38(5):527–35. doi: 10.1016/j.jadohealth.2005.03.021 16635763

[83] Magklara K, Skapinakis P, Niakas D, Bellos S, Zissi A, Stylianidis S, et al. Socioeconomic inequalities in general and psychological health among adolescents: a cross-sectional study in senior high schools in Greece. Int J Equity Health. 2010;9(1):3. 20181002 10.1186/1475-9276-9-3 PMC2837664

[84] Irlbauer-Müller V, Eichler A, Stemmler M, Moll GH, Kratz O. [Parenting stress and the reliability of parental information in the diagnostics of children and adolescents with symptoms of psychiatric and behavioral disorders] Elterliche Belastung und die Zuverlässigkeit von Elternangaben in der Diagnostik psychisch und verhaltensauffälliger Kinder und Jugendlicher. Z Kinder Jugendpsychiatr Psychotherap. 2017;45(4):303–9. German. 10.1024/1422-4917/a000467 27535206

[85] Richter M, Dragano N, Lampert T, Loss J, Pischke C, Schneider S, et al. Understanding the institutional context of health inequalities among young people: Study protocol for a multi-center research unit. Working Paper 001. FOR2723. 2019. SocArXiv. Available from: https://www.for2723.de/en/einblicke.

