# Supplementary material for: Socioeconomic position and self-rated health among female and male adolescents: The role of familial determinants in explaining health inequalities. Results of the German KiGGS study
Source: PLoS One. 2022 Apr 7;17(4):e0266463. doi: 10.1371/journal.pone.0266463 (PMC8989218; doi:10.1371/journal.pone.0266463)
Supplement: S1 Table — *** p < 0.001 ** p < 0.01 * p < 0.05 n.s. not significant. (DOCX) [file pone.0266463.s001.docx]

**S1 Table. P-values for associations between SRH and SEP indicators with familial determinants (results of bivariate linear regression analyses)**

| p-values | **SRH** | | **Income** | | **Education** | | **Occupational status** | | **SEP Index** | | **SSS** | |
| --- | --- | --- | --- | --- | --- | --- | --- | --- | --- | --- | --- | --- |
| *Variable* | *female* | *male* | *female* | *male* | *female* | *male* | *female* | *male* | *female* | *male* | *female* | *male* |
| Family cohesion | *** | *** | *** | * | n.s. | n.s. | *** | n.s. | *** | n.s. | *** | *** |
| Parental well-being | *** | ** | *** | *** | *** | *** | *** | ** | *** | *** | *** | *** |
| Number of stressors | * | * | *** | * | * | n.s. | * | n.s. | *** | n.s. | *** | *** |
| Parenting style (mother) | *** | *** | * | ** | n.s. | *** | * | * | * | *** | *** | ** |
| Parenting style (father) | *** | *** | *** | *** | *** | *** | *** | * | *** | *** | *** | *** |
| Parental smoking | n.s. | * | *** | *** | *** | *** | *** | ** | *** | *** | ** | *** |
| Parental obesity | * | ** | *** | *** | *** | *** | *** | *** | *** | *** | * | n.s. |
| Parental sporting activity | * | n.s. | *** | *** | *** | *** | *** | *** | *** | *** | ** | *** |

*** p < 0.001 ** p < 0.01 * p < 0.05 n.s. not significant
